# Management of a Foreign Body in the Throat: Should Awareness of Channelled Flexible Nasendoscopes Be Encouraged Among Junior ENT Doctors?

**DOI:** 10.7759/cureus.94816

**Published:** 2025-10-17

**Authors:** Siddharth Kotikalapudi, Atanu Maity, Amro Hassaan

**Affiliations:** 1 Otolaryngology, Barking, Havering and Redbridge University Hospitals NHS Trust, London, GBR

**Keywords:** airway foreign body, cost-benefit analysis, ent procedures, fish bone, foreign body retrieval

## Abstract

Ingested foreign bodies in the upper aero-digestive tract are a common ENT emergency. Initial assessment and management by a junior ENT doctor involves removal of the foreign body under direct visualisation, with escalation to removal under general anaesthesia (GA) if unsuccessful. However, removal under general GA carries procedural risks, anaesthetic risks, and a higher cost profile. An adult female presented to our unit with a fish bone lodged in the right pyriform fossa. Initial removal with instruments and direct visualisation with a flexible nasendoscope (without a working channel) was attempted but was unsuccessful. She was scheduled for removal under GA. At morning handover, an alternative approach was suggested (a method not routinely familiar to the on-call junior doctor team) and trialled using a single-use flexible nasendoscope with a working channel. Following topical anaesthesia, the scope was passed orally, and biopsy forceps were introduced via the accessory channel, which allowed for the successful removal of the fish bone. The patient was discharged the same day without complications, avoiding admission and GA. She was followed up with a telephone call and reported that she had returned to her normal routine with no significant complications. While not a novel treatment plan, flexible nasendoscopes with working channels represent a valuable adjunct in escalation protocols for foreign body retrieval. Their use can reduce the need for GA, prevent unnecessary admissions, and optimise resource use in selected cases. Education and training for these instruments can be considered or encouraged among junior ENT doctors to avoid unnecessary admissions for these presentations. However, broad conclusions should not be extrapolated from a single case study, and further audits and surveys of junior doctors’ knowledge and expertise are required.

## Introduction

Ingested foreign bodies within the upper aero-digestive tract are a common ENT emergency presentation, accounting for up to 20% of ENT emergency presentations [1]. In the adult population, fish bones account for a large proportion of these presentations [2]. Emergency referrals are commonly managed by a junior ENT doctor of varying experience; as a result, an unnecessary number of patients are examined under general anaesthesia, with a reported 20% of patients undergoing a theatre procedure where no foreign body is found [1]. Initial management, after it is deemed that there are no airway concerns, is the removal of the foreign body under direct visualisation (single-use flexible endoscope with no working channel). Failure of this method results in escalation for removal of the foreign body under general anaesthesia (GA), which inherently carries its own set of risks [3]. This case study highlights the importance of awareness of a flexible endoscopy with a working channel to remove the foreign body.

## Case presentation

Presentation

A patient had presented overnight with a sensation of a foreign body in her throat, after having dinner involving fish. Upon initial assessment and examination, she was found to be able to eat and drink and did not have any difficulty in breathing. However, she reported a stinging sensation within her throat whenever she swallowed and felt it localise to the right side of her neck (level 2/3). She presented to her local A&E within an hour of the initial onset of these symptoms.

**Figure 1:**
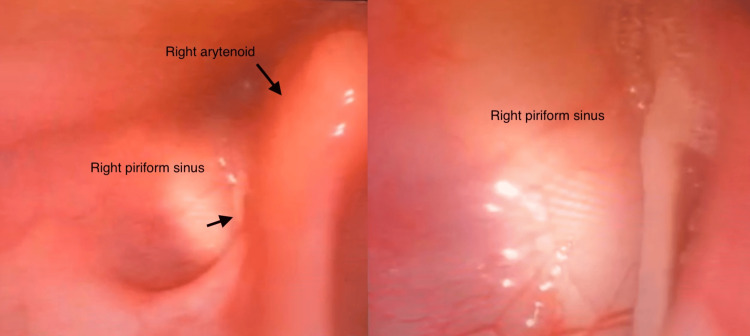
Fish bone in the right pyriform fossa. (a) View of the fish bone from a distance. (b) A close-up view of the fish bone.

There were no immediate concerns for an airway emergency, and the remaining vital signs were normal (SpO_2_, 98%; unremarkable respiratory rate, heart rate, and blood pressure). A flexible nasendoscopy was performed, which visualised a fish bone within the right piriform fossa (see Figure 1) and removal was attempted under direct visualisation by the on-call SHO. However, this was unsuccessful as the fish bone was small (roughly 2 cm) and was in an unfavourable position for removal with Tilley’s or Dennis-Brown forceps under direct visualisation. The patient’s case was discussed with the on-call registrar, who had advised admission and removal under GA in the emergency theatre on the next day.

Management

This presentation was discussed during our morning meeting, and a decision was made by the on-call ENT consultant to attempt removal under direct visualisation again. However, we used a flexible nasendoscope (AmbuScope) with an accessory channel, which would allow for the introduction of biopsy forceps. The larger nasendoscope was introduced after instilling a co-phenylcaine spray via the nostrils. However, despite the local anaesthetic, the larger scope was proving to be difficult to pass through the nasal passage as the patient found this to be quite uncomfortable (complaining of pain, rated 5/10). The scope was alternatively passed via the oral cavity (after further sprays of co-phenylcaine), which the patient was able to tolerate better, and the foreign body was identified in the right pyriform fossa. Biopsy forceps were then introduced via the accessory channel, and the fish bone was removed. The length of the procedure was approximately 20-25 minutes, and she was observed for a further hour until the effects of the spray had worn off. She was monitored for airway compromise, bleeding, or persistent discomfort, and no deterioration was noted during this observation period. She was discharged home with safety netting for any symptoms suggestive of complications. The patient was further followed up with a telephone appointment to discuss her aftercare and return to normal life. She did not report any complications and reported that she returned to her usual routine on the same day, although she voiced that she was cautious of eating fish. Thus, an examination under GA was prevented, and the patient did not require an extended stay in the hospital.

## Discussion

Fish bones are a common ingested foreign body in adults, particularly in populations with high fish consumption [4]. Complications occur when a lodged fish bone or sharp fragment becomes impacted, potentially leading to infection, ulceration, or perforation. In rare cases, patients can present with a retropharyngeal abscess or mediastinitis, which carries significant morbidity and mortality [5].

Initial assessment of a patient with a suspected foreign body in the throat involves excluding airway compromise. Once a safe airway is confirmed, flexible nasendoscopy is the most reliable modality to confirm the presence of a foreign body [6], as radiographs often lack sufficient sensitivity for radiolucent fish bones [7,8]. If a foreign body is not identified on flexible nasendoscopy or radiographs, and symptoms persist, CT imaging of the neck is warranted.

If initial removal attempts fail, a further attempt using a larger endoscope with an accessory channel may be considered. This approach can prevent hospital admission and avoid examination under GA with rigid oesophagoscopy or laryngoscopy, which carry inherent risks, including mucosal tears, bleeding, oesophageal perforation, and mediastinitis [9]. Additional risks include perioperative complications and prolonged recovery, particularly in comorbid or elderly patients [9]. Anaesthetic risks, such as airway manipulation, intubation complications, and drug-related adverse effects, must also be considered, with rarer risks including laryngospasm, aspiration, or difficult intubation [10,11].

Existing evidence supports flexible endoscopy as an effective alternative to rigid endoscopy. A meta-analysis of 1,402 patients reported equivalent success rates for flexible endoscopy and rigid endoscopy (pooled odds ratio (OR) = 1.00, 95% confidence interval (CI) = 0.48-2.06), with low overall complication rates; the pooled estimate suggested a higher, though not statistically significant, risk of perforation with rigid endoscopy [9]. These data suggest that flexible endoscopy with appropriate instrumentation may serve as a safe and cost-effective first-line or intermediate approach in selected patients, potentially avoiding GA in many cases. However, as the meta-analysis primarily evaluated oesophageal impactions, extrapolation to pharyngeal or hypopharyngeal foreign bodies must be done cautiously. There remains a lack of direct comparative data between rigid and flexible techniques for pharyngeal or hypopharyngeal retrievals, and current clinical guidelines do not differentiate between standard flexible nasendoscopy and larger flexible nasendoscopy with a working channel [12]. This underscores the need for further research and structured evaluation of flexible endoscopy in these anatomical regions.

Patients undergoing foreign body retrieval under local anaesthetic generally do not require extended observation or hospital admission. Procedures are typically less invasive, with lower risk profiles, and patients can often be discharged on the same day with appropriate safety netting [9-11]. Nonetheless, without larger series or control groups, these benefits remain observational.

Flexible endoscopy with a working channel does require at least two experienced clinicians. Patient discomfort during transnasal endoscopy may reduce cooperation and success rates, and adequate topical anaesthesia may not fully alleviate this [13,14]. In our case, passing the endoscope orally was trialled and was successful; however, this is less common and should be undertaken only by experienced clinicians. Current literature on transoral applications of single-use Ambu® aScope™ devices is limited, indicating a need for further research and clinical evaluation before widespread adoption.

This approach is unsuitable for foreign bodies that have passed into the oesophagus, which typically require GA and rigid endoscopy. Success also depends on patient tolerance, anatomical factors such as narrow nasal passages, and the size of the foreign body. In these scenarios, escalation to GA remains the safest approach.

The cost-benefit analysis of foreign body retrieval under local anaesthesia compared to foreign body retrieval under GA must be considered. The unit price of a single-use endoscope with a working channel is £179 (excluding VAT) [15] and can avoid admission or an overnight stay in the hospital, if the procedure is successful. It also avoids assessment and workup from the anaesthetic team and theatre occupation, which allows for other urgent presentations that require GA and eliminates reprocessing, repair, and cross-contamination risk of space and equipment [14]. Table 1 presents a comparison of the benefits and risks associated with the removal under GA and removal with a single-use endoscope.

**Table 1:** Comparison of costs and benefits of removal under GA and removal with a single-use flexible endoscope. Costs for failed FNE assume escalation to GA; actual cost may vary depending on theatre scheduling and hospital protocols. Savings assume successful retrieval on first FNE attempt. FNE = flexible nasendoscopy; GA = general anaesthesia

Factor	GA retrieval	Single-use flexible endoscope retrieval
Theatre cost	£840–£1,260 (60–90 minutes)	N/A
Admission cost	£350–£587 per day (pre/postoperative + overnight stay)	Admission normally not required
Equipment cost	A reusable rigid scope which requires reprocessing and potential repair can lead to high life cycle costs (£104.92 to £209.84 per endoscope) [15]	£179 (excluding VAT). Single use biopsy forceps: £9.22–12.50 (variable between different manufactures)
Anaesthesia risk	Complications from GA are applicable	N/A; only a local anaesthetic is used
Staffing demands	All theatre staff (anaesthetist surgeon, scrub nurse, ODP, etc.)	ENT specialist and an assistant
Recovery time	Monitoring after GA, admission, and monitoring	Same-day discharge
Cross-contamination risk	Present in reusable scopes	None present in single-use scopes

**Figure 2:**
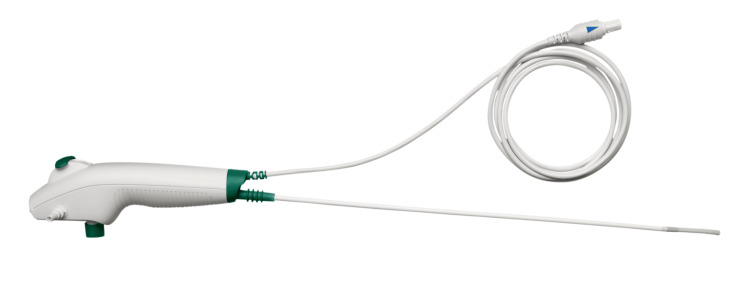
Ambu A/S (Ambu® aScope™ 4 RhinoLaryngo Intervention). Image courtesy of Ambu A/S. Used in accordance with Ambu’s media policy [19].

Direct costs of admission for removal of foreign body under general anaesthesia are as follows: (1) these cases are often booked for a typical theatre time of 60-90 minutes (including anaesthetic time, setup, and turnover); the cost would range from £840-1,260 (£14/minute) [16,17]; (2) the cost of admission into hospital, including pre- and postoperative waiting time: £350-£587 per day [18,19]; and (3) displacement of other emergency cases, which can have large system costs [16]. Figure 2 presents a representative image of the single-use Ambu® aScope™ device.

Therefore, any case that occupies the theatre for more than 13 minutes has a higher cost than the use of a single-use channelled scope. Therefore, savings per successful removal with a single-use endoscope can range from £998.50 to £1,655.50 (Table 2).

**Table 2:** Savings per case when comparing single-use endoscope retrieval versus GA retrieval of foreign body. Costs in GBP (£). FNE failure assumes escalation to GA, eliminating financial benefit. Does not include indirect system costs (e.g., displaced emergency cases). FNE = flexible nasendoscopy; GA = general anaesthesia

Scenario	FNE cost (£)	GA total cost (£)	Savings (£)
FNE success – lower estimate	191.50	1,190.50	998.50
FNE success – upper estimate	191.50	1,847	1,655.50
FNE failure → GA	191.50 + GA cost (1,190.5/1,847)	1,190.50–1,847	0

Case selection is vital in these presentations as in patients with airway compromise, foreign bodies in unfavourable positions (such as oesophagus) will likely require GA and rigid endoscopy [1,4]. The use of single-use endoscopes for retrieval in these cases is unsafe and can double the costs when they are required to go to the theatre. The cost analysis from this case study suggests that a successful retrieval with a flexible endoscope can be economically beneficial, but these estimates assume procedural success. Failed attempts requiring escalation will double the costs. A sensitivity analysis and an audit of larger case series are needed to validate the cost-effectiveness.

Systemic limitations affect the potential adoption of using channelled flexible endoscopes among junior doctors. Lack of access and knowledge of the equipment affects junior doctors’ confidence and competency in challenging endoscopic manipulation. Limited training and access may increase failed attempts, leading to GA escalation and higher risk and cost. Implementing structured training programs for junior ENT doctors can enhance proficiency in flexible endoscopy techniques and increase awareness of the different types of endoscopes available. Regular audits of procedural outcomes, once these techniques are in routine use, will help identify areas for improvement and ensure appropriate and effective application. However, there is currently a lack of national survey data assessing junior doctors’ familiarity and use of working-channel flexible endoscopes. Robust national data are required to inform the need and design of future training programmes.

## Conclusions

This case study helps illustrate that a working-channel flexible endoscopy can be used to safely retrieve foreign bodies under topical anaesthesia. Examination under anaesthesia, admission, and retrieval of the foreign body in controlled settings, such as a theatre, can be avoided. However, as a single-case observation, the findings cannot be extrapolated. Success depends on patient selection, anatomical considerations, and clinician familiarity with such techniques. This highlights the potential value of structured training programs, competency assessments, and regular audits for junior doctors. Further prospective studies and cost-benefit analysis are warranted to guide broader adoption and protocol development. Current literature comparing flexible and rigid endoscopy removal specifically in the pharynx or hypo-pharynx is limited, and current guidelines do not differentiate between standard and working-channel flexible nasendoscopy, which raises the question of whether they should be differentiated in future guidelines.
